# *In vitro* antimicrobial activity of a novel compound, Mul-1867, against clinically important bacteria

**DOI:** 10.1186/s13756-015-0088-x

**Published:** 2015-11-06

**Authors:** George Tetz, Victor Tetz

**Affiliations:** TGV-Therapeutics, Institute of Human Microbiology, LLC, 303 5th avenue, suite 2012, New York, NY 10016 USA; Department of Microbiology, Virology, and Immunology, Pavlov First St. Petersburg State Medical University, St. Petersburg, Russia

**Keywords:** Bactericidal, Biofilms, Resistant, Time-kill, Chlorhexidine gluconate

## Abstract

**Background:**

The antimicrobial activity of Mul-1867, a novel synthetic compound, was tested against 18 bacterial strains, including clinical isolates and reference strains from culture collections.

**Methods:**

The minimal inhibitory concentration (MICs) and minimal bactericidal concentration (MBCs) were determined by using the broth macrodilution method. The kinetics of the inhibitory effects of Mul-1867 against biofilm-growing microorganisms was assessed at time-kill test *in vitro* against 48-h-old biofilms of *Staphylococcus aureus* and *Escherichia coli*. Transmission electron microscopy analyses was conducted to examine cell disruption.

**Results:**

A comparative assessment of the antimicrobial activities of Mul-1867 and chlorhexidine digluconate (CHG), used as a control antimicrobial, indicated that Mul-1867 was significantly more effective as a disinfectant than CHG. Mul-1867 showed potent antimicrobial activities against all the tested bacteria (MIC: 0.03–0.5 μg/mL). Furthermore, MBC/MIC ratio of Mul-1867 for all tested strains was less than or equal to 4. Time-kill studies showed that treatment with Mul-1867 (0.05–2 %) reduced bacterial numbers by 2.8–4.8 log10 colony forming units (CFU)/mL within 15–60 s. Bactericidal activity of Mul-1867 was confirmed by morphological changes revealed by TEM suggested that the killing of bacteria was the result of membrane disruption.

**Conclusion:**

Overall, these data indicated that Mul-1867 may be a promising antimicrobial for the treatment and prevention of human infections.

## Background

Microbial infections are one of the main causes of morbidity and mortality. Infectious disease treatments are associated with challenges including increasing antimicrobial resistance, drug cytotoxicity, and limited drug spectrum and these difficulties have instigated novel antimicrobial drug development [1–3]. Antibiotic and antiseptic resistance have partly emerged by the prevalence of bacteria in the form of biofilms, which enable microorganisms to survive antibiotic concentrations that are 1000 times higher than the minimal inhibitory concentration (MIC) [4–6]. Previous studies have attributed the high antibiotic tolerance of biofilms to a lipid-composed film that is present on the outer surface and due to the presence of extracellular polymeric substances inside microbial community [7, 8]. Biofilms have also been associated with a variety of human infections and are known to be poor responders to antibiotic and antiseptic therapy [9–11].

Topical antimicrobial agents are routinely used in various branches of medicine such as dentistry, otolaryngology, surgery and gynecology [12–16]*.* They are also used to reduce nosocomial infections and are particularly useful in preventing infections in intensive care unit patients [17–19]. Chlorhexidine gluconate (CHG) is one of the most widely used antimicrobial agents due to its broad spectrum of antimicrobial action and compatibility with several types of materials [20–23]. The above-mentioned properties of CHG have enabled its incorporation in numerous pharmaceutical and medical devices [24, 25].

Furthermore, the emergence of chlorhexidine-resistant microorganisms, for example *Klebsiella pneumoniae* and *Staphylococcus aureus* have also posed treatment challenges [26, 27]. Thus, there is a critical need for the development of new antimicrobials that have a broad-spectrum of antimicrobial activity and that can be effectively used to treat resistant bacteria.

**Fig. 1:**
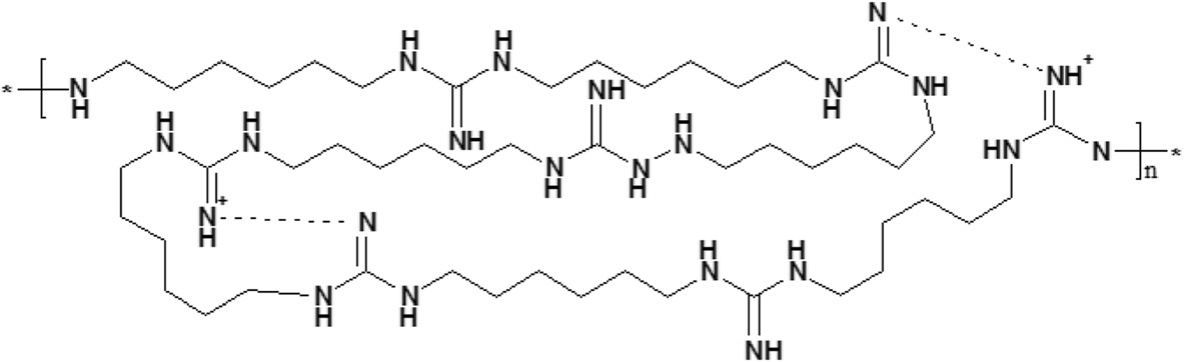
Chemical structure of Mul-1867, *n* = 1–20

In this study, we describe a potential new antimicrobial compound called Mul-1867 [poly-N1-hydrazino(imino)methyl-1,6-hexanediamine; Fig. 1].

The objective of this study was to assess the *in vitro* antibacterial activity of Mul-1867. Furthermore, the comparative antimicrobial efficacies of Mul-1867 and CHG against established 48-h-old biofilms were also assessed.

## Methods

### Antimicrobial agents

Mul-1867 was synthesized in TGV-Laboratories Inc. (NJ, USA). CHG solution (20 % in H_2_O) was obtained from Sigma Chemical Co. (St. Louis, Mo.). Mul-1867 and CHG were diluted with sterile distilled water on the day of use.

#### Bacterial strains

*Bacillus cereus* VT-289, *Enterococcus faecalis* VT-72, *E. faecalis* VT-693, *Escherischia coli VT-1402, K.pneumoniae VT-*1367, *Neisseria subflava* VT-455, *Pseudomonas aeruginosa VT-177, Salmonella enterica* VT-191, *S. аureus* MSSA VT 961, *S. aureus* MRSA VT −234, *Streptococcus epidermidis* VT-432, *S. mitis* VT-842, and *S. pyogenes* VT 59, *S. epidermidis* VT 908 were obtained from a private collection (provided by Dr. V. Tetz, Institute Of Human Microbiology, LLC). Control strains included *E. faecalis* ATCC 29212, *E. coli* ATCC 25922, *P. aeruginosa* ATCC 27853, and *S. aureus* ATCC 29213 and were purchased from American Type Culture Collection (ATCC). All bacterial strains were subcultured from freezer stocks onto brain heart infusion agar plates (BHI; Difco, Detroit, MI, USA) and incubated at 37 °C overnight. All subsequent liquid subcultures were derived from colonies isolated from these plates and were grown in Luria-Bertani broth medium (LB, Oxoid).

### Antimicrobial susceptibility of planktonic bacteria

The MICs and minimal bactericidal concentrations (MBCs) for antimicrobials were determined by using the broth macrodilution method in accordance with the Clinical and Laboratory Standards Institute guidelines [28, 29]. A standard inoculum of 5 × 10^5^ CFU/mL was used. Serial 2-fold dilutions of the antimicrobials were prepared in cation-adjusted Mueller-Hinton broth. The MIC was defined as the lowest concentration of antibiotic that completely inhibited visible growth. The MBC was defined as the lowest antimicrobial concentration that killed ≥ 99.9 % of the initial bacterial count (≥3 log10 CFU/mL) in 24 h.

### Kinetics of Mul-1867 and CHG activity against biofilms

To determine the minimum concentration and exposure time required by Mul-1867 and CHG to kill biofilms, we performed a time-kill test *in vitro* and assessed the activity of serially diluted Mul-1867 and CHG against the biofilms of *S. aureus* ATCC 29213 and *E. coli* ATCC 25922. This method was developed for determining MIC of antibiotics against planktonic growing cells by Ernst et al. [30]. We used a modified protocol of this method to study the antimicrobial activities of Mul-1867 and CHG against preformed biofilms. Briefly, all bacterial cultures were grown overnight as liquid cultures and 200 μL inoculum (5 × 10^5^ CFU/mL) for each strain was transferred to a 96-well microtiter plates (Sarstedt, Numbrecht, Germany). The plates were then incubated for 48 h at 37 °C. The wells proximal to the plate frame were filled with medium only and were used as negative controls for growth. After incubation, the growth medium was removed from the wells without disrupting the integrity of the biofilms. The formed biofilms were then gently washed three times with PBS to remove nonadherent cells. Next, 200 μL of Mul-1867 or CHG diluted in sterile distilled water were added in the appropriate wells for 15, 30, or 60 s. Untreated biofilms were used as negative controls for each isolate at each time point. After the exposure, well contents were aspirated again and to prevent antimicrobial carry-over each well was washed three times with deionized water. Biofilms were scraped thoroughly, with particular attention to well edges. The well contents were aspirated again, then placed in 1 mL of PBS and the total CFU number was determined by serial dilution method and plating on appropriate media. Data were converted to a log10 scale and compared to 1 x 10^8^ CFU untreated 48-h-old biofilms. All assays included at minimum 2 replicates and were repeated in 3 independent experiments.

### Transmission electron microscopy

Transmission electron microscopy analyses was conducted to examine cell disruption. The assay was performed using planktonic growing *S. aureus* ATCC 29213. Bacteria were centrifuged 3000 g (Eppendorf 5415 C centrifuge; Eppendorf Geratgebau GmbH, Hamburg, Germany) and suspended in isotonic phosphate buffer (0.15 M, pH 7.2). Mul-1867 was added to the bacterial suspension to a final concentration of 0.5 %. Tubes were shacked for 30 s at 22 °C and bacteria were harvested by 10 min of centrifugation at 3000 g. After treatment, cells were observed under a transmitting electron microscope (Carl Zeiss, LIBRA 120) at 80 kV [31, 32].

### Evaluation of Mul-1867 effect on membrane integrity

The assay was performed using planktonic growing *S. aureus* ATCC 29213 and *E. coli* ATCC 25922*.* Mul-1867 was added at final concentration 0.5 % to a standard inoculum of 5 × 10^7^ CFU/mL. Tubes were shacked for 30 s at 22 °C and bacteria were harvested by 10 min of centrifugation at 3000 g (Eppendorf 5415 C centrifuge). DNA was extracted from the supernatant solution with QIAamp DNA Mini Kit (Qiagen, GmbH), following the manufacturer’s instructions. DNA amount was determined by optical density at 260 nm (OD260) using an Eppendorf BioPhotometer 6131 (Hamburg, Germany). To control the purity of the DNA, only samples with an OD260/OD280 ratio of 1.8–2.0 were used for subsequent analysis. A background value of OD at 320 nm was subtracted from the measured values of OD260 and OD280. Measurements were conducted at room temperature.

### Statistical analysis

All experiments were performed in triplicates. Results are provided as mean ± standard deviation. All statistical analyses were performed using the statistics package Statistica for Windows (version 5.0). A *p*-value of < 0.05 was considered statistically significant.

## Results

### Antibacterial activity

The MICs and MBCs of Mul-1867 against 18 bacterial strains are shown in Table 1.

**Table 1:** *In vitro* antibacterial activity of Mul-1867

Organism	Mul-1867 (mg/L)		CHG (mg/L)	
	MIC	MBC	MIC	MBC
Gram-positive bacteria				
* B. cereus* VT-289	0.5	2	1.0	2.0
* E. faecalis* ATCC 29212	0.03	0.06	2.0	8.0
* E. faecalis* VT-72	0.06	0.125	4.0	16.0
* E. faecalis* VT-693	0.06	0.06	2.0	16.0
* S. aureus* АТСС 29213	0.125	0.15	2.0	2.0
* S. аureus* MSSA VT-961	0.06	0.125	2.0	2.0
* S. aureus* MRSA VT-234	0.06	0.125	4.0	4.0
* S. epidermidis* VT-432	0.06	0.125	2.0	16.0
* S. epidermidis* VT 908	0.25	0.5	2.0	16.0
* S. mitis* VT-842	0.06	0.06	4.0	64.0
* S. pyogenes* VT 59	0.125	0.125	8.0	16.0
Gram-negative bacteria	ᅟ	ᅟ	ᅟ	ᅟ
* E. coli* АТСС 25922	0.25	0.25	2.0	16.0
* E. coli* VT-1402	0.06	0.06	16.0	32.0
* K. pneumoniae* VT-1367	0.125	0.25	32.0	32.0
* N. subflava* VT-455	0.125	0.25	8.0	8.0
* P. aeruginosae* ATCC 27853	1.0	1.0	32.0	64.0
* P. aeruginosae* VT-177	0.125	0.25	32.0	32.0
* S. enterica* VT-191	0.5	2	16.0	32.0

Mul-1867 inhibited the growth of all the microorganisms tested, with MIC values ranging from 0.03 to 0.5 mg/L. The MIC values of Mul-1867 against bacteria were considerably lower than those of CHG. Notably, Mul-1867 antibacterial activity was 2 to 256 times higher than that of CHG which MIC values ranging from 1 to 32 mg/L. The most resistant test organisms, *B. cereus* and *S. enterica*, were killed by 2 mg/L of Mul-1867. In contrast, list the most resistant organisms to CHG were *K. pneumonia* and *P.aeruginosa* with MIC 32 mg/L. The MBCs values of Mul-1867 were lower than 0.5 mg/L for 14 strains, and the MBC values for the other 4 strains ranged from 0.5 to 2 mg/L. After Mul-1867 treatment, the MBC/MIC ratios of 86 % microbial strains were < 4, whereas, those of the remaining 14 % of cultures were 4. Thus Mul-1867 inhibited bacterial growth of all bacterial strains in a bactericidal manner (MBC/MIC ratio ≤ 4). After CHG treatment, the MBC/MIC ratios were < 4 for 60 % strains and the ratios ranged from 4 to 12 for the remaining 40 % strains indicating that CHG displays bactericidal and bacteriostatic effects. No Mul-1867-resistant strain was identified among the clinical isolates.

### Kinetics of inhibitory effect of Mul-1867 against bacterial biofilms

**Fig. 2:**
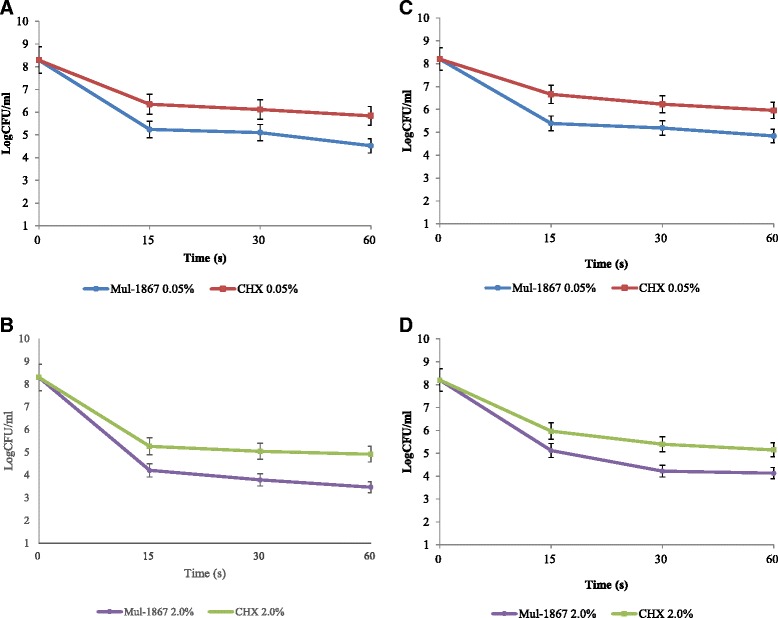
*In vitro* time-kill assessment of the bactericidal activity of Mul-1867 and CHG against bacterial biofilms. Kill curves of (**a**, **b**) *S. aureus*, (**c**, **d**) *E. coli* biofilms exposed to various concentrations (0.05 and 2 %) of either Mul-1867 or CHG for various durations (15, 30, and 60 s) are shown. The antiseptic inhibitory effect on biofilms was assessed by measuring the number of CFU obtained after antibiotic treatment. All experiments were performed in triplicates

The kinetics of the inhibitory effects of Mul-1867 against biofilm-growing microorganisms are summarized in Fig. 2 (*p* < 0.005).

Mul-1867 or CHG displayed a concentration-dependent bactericidal activity against 48-h-old biofilms and exhibited a significant reduction in the number of viable bacteria within biofilms for all periods of exposure. There was a dose-dependent decrease in CFU numbers with increase in antibiotic exposure time for antimicrobials at concentrations of 0.05 % or higher. Our study demonstrated that both antiseptics possess microbicidal activity that can be effective within contact times ranging from 15 to 60 s. The number of viable cells dropped dramatically after 15 s, after which cell death continued at a much slower rate. The bactericidal efficiency of Mul-1867 was higher than that of CHG at the same concentrations. We found that Mul-1867 was approximately up to 20 times more efficient than CHG at all time points assessed.

### Mode of action of Mul-1867 on bacteria

**Fig. 3:**
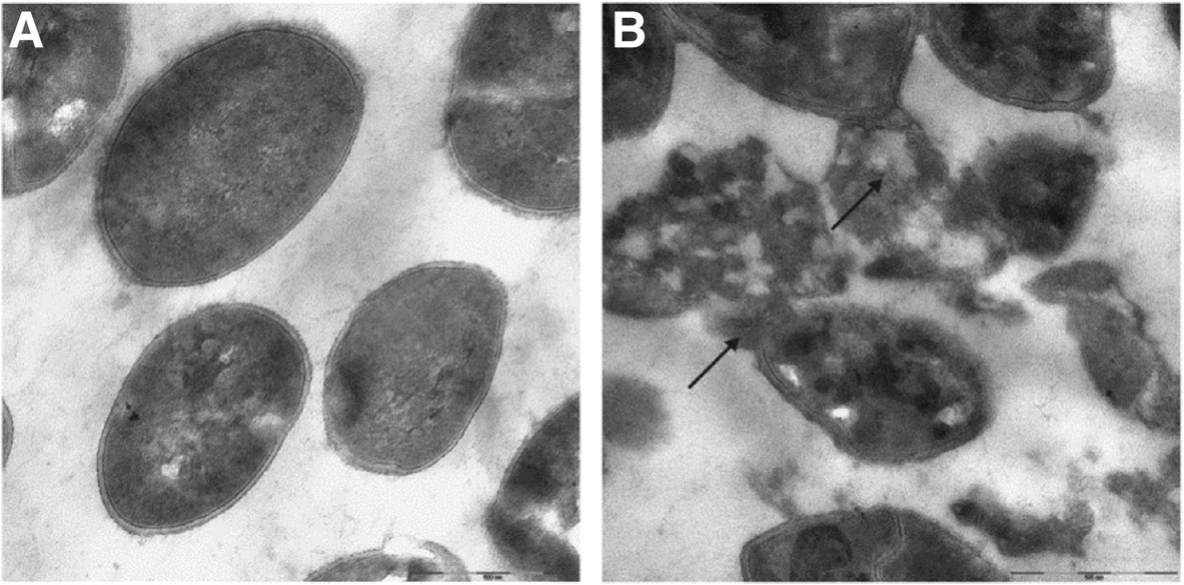
TEM micrographs of (**a**) untreated *S.aureus* (**b**) incubation with a 0.5 % Mul-1867 for 30 s shows some completely lysed cells

The untreated *S.aureus* cells, prepared for TEM micrographs shown a normal cell shape with an undamaged structure of the inner membrane (Fig. 3a). In all cases bacteria appeared to be lysed after Mul-1867 treatment (Fig. 3b).

The images of *S.aureus* strains show that treated bacteria are collapsed and their cell walls are degraded. The black arrows point out the dead cells with damaged membranes accompanied by the leakage of intracellular components.

Among the indicators of membrane damage, an increased DNA concentration from lysed bacteria in the medium measured spectrophotometry at OD260 was detected (Table 2).

**Table 2:** Effect of Mul-1867 on DNA release from bacteria

Probe	OD260	
	Untreated	Exposed to 0.5 % Mul-1867
*E.coli*	0.058	0.238
*S.aureus*	0.062	0.279

The amount of DNA was increased up to 4.5 times following Mul-1867 treatment for 30 s compared to untreated cells.

## Discussion

In this study, we examined the antibacterial activitiy of both Mul-1867 and CHG against clinical isolates and reference strains. We have shown that Mul-1867 had broad-spectrum, fast-acting microbicidal activity. It seems to act like other members of the polymeric guanidine family targeting the membrane of the microorganisms [33].

In this study, Mul-1867 was shown to exhibit high antimicrobial activity against all tested strains, including MRSA, which were previously shown to respond poorly to treatment with existing medicines [34, 35]. Importantly, we found that the MICs and MBC values of Mul-1867 were less than 4 against a variety of gram-positive bacteria, gram-negative bacteria, indicating that Mul-1867 possesses bactericidal activity [28, 36]. Bactericidal activity of Mul-1867 was confirmed by morphological changes revealed by TEM suggested that the killing of bacteria was the result of membrane disruption. It is confirmed by an increased amount of DNA in the medium 30 s after Mul-1867 treatment. The molecule of Mul-1867 contains guanidine and hydrazine derivatives. It is known that guanidines groups binds to negatively charged molecules on bacterial surface like carboxyl group (−COOH) of the fatty acid and hydrazine react with carbonyl groups [37, 38]. Binding of guanidine and hydrazine groups to phospholipids, cause bacterial death, followed by disruption of the cell wall and consequent bacteria lysis [39].

The main limitation of MIC and MBC measurements is the inability of the method to determine the rate of microbicidal activity. In our studies, we assessed bacterial viability against Mul-1867 and CHG with time-kill determinations [40]. This method determines the viability of the organisms after contact with antimicrobials for a specified time period and allows us to evaluate the effects of the potential antimicrobial on biofilms, which play a significant role in numerous human diseases and contribute to treatment inefficiency [41–43]. Time-kill studies were performed with 48-h-old biofilms, and the antimicrobial effects of both Mul-1867 and CHG were determined using two commonly used chlorhexidine concentrations [44, 45]. Time-kill curves of the isolates have shown a clear relationship between the extent of inhibition and the concentrations of Mul-1867 and CHG, indicating that both compounds possessed dose-dependent microbicidal activity and could be effective within contact times starting at 15 s. Importantly, Mul-1867 was up to 20 times more efficient than CHG at all time points assessed.

## Conclusions

Taken together, our data suggested that Mul-1867 is a promising novel antimicrobial agent that has potent broad-spectrum antibacterial activity against clinically important microorganisms. Further studies will be directed towards development of Mul-1867 as a locally acting antimicrobial.
